# Puberty is a Critical Period for Vomeronasal Organ Mediation of Socio-sexual Behavior in Mice

**DOI:** 10.3389/fnbeh.2020.606788

**Published:** 2021-01-22

**Authors:** Sarah K. J. Cross, Yellow H. Martin, Stephanie Salia, Iain Gamba, Christina A. Major, Suhail Hassan, Katelyn A. Parsons, Ashlyn Swift-Gallant

**Affiliations:** Department of Psychology, Memorial University of Newfoundland, St. John’s, NL, Canada

**Keywords:** vomeronasal organ (VNO), puberty, social behaivor, aggression, sexual behavior, critical period, odor preference

## Abstract

Genetic disruption of the vomeronasal organ (VNO), an organ responsible for pheromone processing, drastically alters socio-sexual behavior in mice. However, it is not known whether the VNO has a role during the pubertal organizational period when sex-typical socio-sexual behaviors emerge, or if disruption of the organ in adulthood is sufficient to alter socio-sexual behavior. To bypass the lifelong VNO disruption of genetic knockout models, we surgically ablated the VNO of male and female mice either during the peripubertal period [postnatal day (PND) 28–30] or adulthood (PND 58–60), with sham controls at both ages. We ruled out anosmia *via* the buried food test and assessed sexual odor preferences by simultaneously exposing mice to same- and opposite-sex soiled-bedding. We then measured territorial aggression with the resident-intruder paradigm and assessed sexual behavior in response to an encounter with an estrus-induced female. Neural activity approximated by FOS-immunoreactivity along the VNO-accessory olfactory pathway was measured in response to opposite-sex odors. We found that peripubertal VNO ablation decreased sexual odor preferences and neural activity in response to opposite-sex odors, and drastically reduced territorial aggression in male mice. Conversely, adult VNO ablation resulted in subtle differences in sexual odor preferences compared with sham controls. Regardless of the VNO condition, mice displayed sex-typical copulatory behaviors. Together, these results suggest that puberty is a critical period in development whereby the VNO contributes to the sexual differentiation of behavior and neural response to conspecific odors.

## Introduction

Pheromonal cues are an important form of communication for many animals (Dulac and Wagner, 2006). These chemical signals transmit social and sexual information involved in conspecific recognition, mate selection, threat detection, and parental care. Upon detection, pheromones can elicit predetermined behavioral and endocrine responses. Rodents have chemosensory neurons within the nasal pathway in both the vomeronasal organ (VNO) and Main Olfactory Epithelium (MOE), though it is the VNO that is largely responsible for processing pheromonal information. The VNO sends its axons to the Accessory Olfactory Bulb (AOB) which then projects to the limbic system and hypothalamic nuclei which are associated with reproductive, aggressive, and parental behaviors (reviewed in Baum, 2009).

Genetic disruption of the VNO *via* knockout of the transient receptor potential 2 ion channel (*Trpc2*) reportedly leads to a sex-reversal in many behaviors. *Trpc2* is highly expressed in the VNO, with little expression elsewhere (Liman et al., 1999), and is critical to the functioning of the VNO (Stowers et al., 2002). *Trpc2*^−/−^ male mice fail to display aggression towards male intruders and instead show copulatory behaviors (e.g., mounting and thrusting) towards both males and females (Leypold et al., 2002; Stowers et al., 2002). *Trpc2*^−/−^ males also display a decreased preference for female odors, even though *Trpc2*^−/−^ males can still discriminate between the sexes (Beny and Kimchi, 2016). *Trpc2*^−/−^ females also show a drastic change in behavior, showing male-typical copulatory behaviors (i.e., mounting and thrusting) toward both male and female stimulus mice (Kimchi et al., 2007).

However, there are mixed results concerning the effects of adult surgical ablation of the VNO (VNOx). In contrast to a constitutive genetic knockout, which pervades the entire life of the animal, surgical ablation has been used in adult mice to evaluate the role of the VNO in adulthood for socio-sexual behavior. Kimchi et al. (2007) reported that surgical ablation in adulthood produces a similar sex-reversal in behavior to genetic ablation (i.e., *Trpc2*^−/−^). They also concluded that since there are only slight differences in the behavior of VNOx and *Trpc2*^−/−^ female mice, VNO input has only a minor impact during development for socio-sexual behavior. In contrast, Martel and Baum (2009) report that adult VNO ablation is insufficient to produce male-typical sexual behavior in female mice, suggesting that the role of the adult VNO is not critical to sex-typical behavior. Given these inconsistencies, the role of the adult VNO remains unclear.

One possibility for the discrepancies between genetic ablation and adult surgical ablation of the VNO is that the VNO in development has long-lasting effects on the brain and behavior. Indeed, there is support for the idea that pheromones facilitate the onset of socio-sexual behavior during the pubertal period (Kaneko et al., 1980; Vandenbergh, 1983), and thus the VNO may be required during puberty when these behaviors emerge.

In the present study, we assessed whether VNO functioning during reproductive maturation is required for the organization of behavior. Given that puberty marks the onset of many socio-sexual behaviors, we hypothesized that puberty may serve as a critical window for VNO mediation of such behaviors. To test this hypothesis, we ablated the VNO of male and female mice during the peripubertal period or in adulthood and compared the sexual and aggressive behavior of these mice in adulthood compared to sham-operated male and female control mice. We then evaluated a marker of neural activity (FOS) along the vomeronasal-accessory olfactory pathway in response to opposite-sex odors.

## Materials and Methods

### Animals

**Figure 1:**
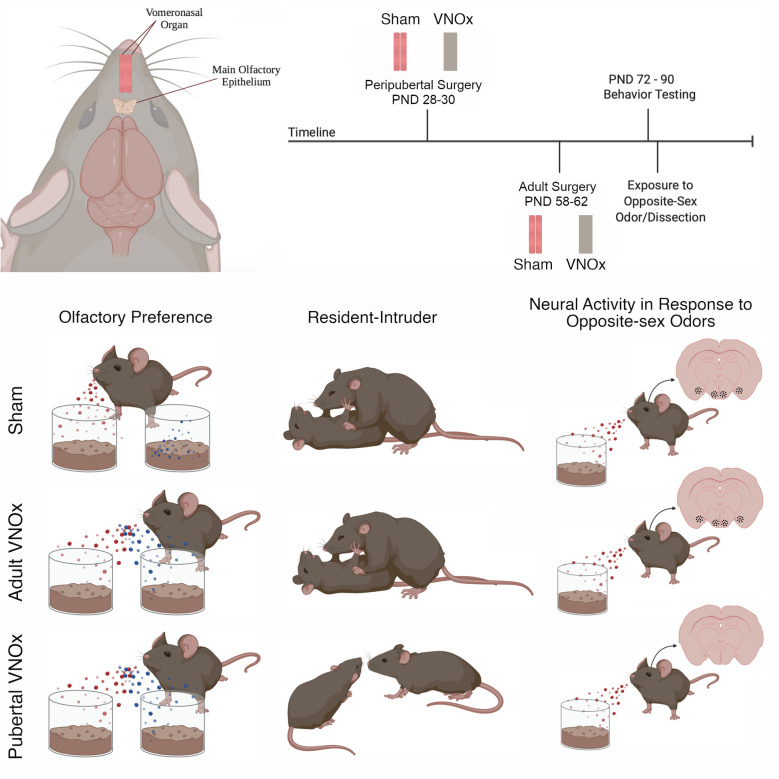
Timeline, procedure, and summary of results. Male and female mice underwent surgical vomeronasal organ ablation (VNOx) or a sham procedure in peripuberty [postnatal day (PND) 28–30] or in adulthood (PND 58–62). Behavioral testing took place between PND 72–90 and included the buried food test, olfactory preference, resident-intruder paradigm, and sexual behavior test. Following behavior testing, mice were exposed to soiled-bedding from the opposite-sex, then brains were dissected 90-min later. One series of brain tissue was processed for FOS immunoreactivity (ir), a marker of neural activity, and a second series was Nissl stained to assess neuroanatomical features, including cell number and size, along the accessory olfactory pathway. We found that peripubertal VNOx decreased preference for female-soiled bedding, reduced territorial aggression in male mice, and decreased FOS-ir in response to opposite-sex odors. Conversely, adult VNOx resulted in subtle differences in sexual odor preferences compared with sham controls but did not alter aggression or FOS-ir in response to opposite-sex odors. Regardless of VNO condition, sex-typical copulatory behaviors were observed, and no differences in neuroanatomy were found. Figure created with Biorender.com.

Twenty-four (12 male, 12 female) C57Bl/6 mice were obtained from Charles River, QC, Canada and paired with opposite-sex conspecifics for breeding. Offspring were weaned at 21–22 days of age. All experimental mice were singly housed between 28–36 days of age and sexually naïve at the onset of behavioral testing. Male and female mice from the same litter were divided among four conditions: Peripubertal sham (*n* = 21; 11 female), peripubertal VNOx (*n* = 22; 10 female), adult sham (*n* = 21; 10 female) and adult VNOx (*n* = 19; 9 female). Peripubertal surgeries took place between 28–30 days of age, while adult surgeries occurred when animals were 58–62 days of age (see Figure 1 for timeline and summary of procedure/results). The timing of peripubertal ablation (28–30 days of age) was selected to coincide with the onset of puberty/before sexual maturity; specifically, GnRH initiates the pubertal endocrine changes and occurs before 28 days of age in mice (Gore et al., 1999) and the androgen surge which is largely responsible for the organization of the brain and behavior (i.e., especially aggression/sexual behavior) begins after 30 days of age (e.g., Edwards, 1970). Vaginal opening, one of the earliest markers of pubertal onset in females, occurs on average at 25 days of age (Ismail et al., 2011), while the balano-preputial separation, a marker of pubertal onset in males, occurs on average at 27–28 days of age in in C57bl/6 mice (Zhou et al., 2007; Molenhuis et al., 2014). Thus, the earliest signs of puberty are shown before 28 days of age in mice, and the major endocrine event underlying male-typical sexual and aggressive behaviors (i.e., androgen surge) occurs following 30 days of age.

Food and water were provided *ad libitum* and animals were kept on a 12-h light-dark cycle with lights on at 7 AM. All procedures outlined follow the Canadian Council on Animal Care (CCAC) Guidelines and were approved by the Institutional Animal Care Committee at the Memorial University of Newfoundland.

### VNO Surgery

Mice were anesthetized with 1–2% isoflurane in a nose cone, supine, and their mouth was held open with forceps during the procedure. An incision was made at the tip of the incisive papilla, and the hard palate was carefully pulled back, exposing the VNO. In sham-operated animals, the VNO was left intact, and the palate was secured back in place with tissue adhesive. In VNOx groups, once the VNO was exposed, an incision was made in the caudal vomer bone and the ventral end of the VNO. Forceps were placed beneath the VNO and the bilateral organ was extricated; if there was resistance in removing the organ, forceps were run along the ventral to caudal VNO, gently pulling up to detach any connective tissues (see Brechbühl et al., 2011 and Doyle et al., 2014 for video graphics depicting the visualization and extrication of the VNO in rodents). The cavity was filled with gel foam (*Pfizer*, GELFOAM, absorbable gelatin sponge) and pressure was applied to control bleeding. Tissue adhesive secured the hard palate back in place. Using a small transfer pipet, the nasal cavity was rinsed with saline until the drained fluid was clear of any blood. Slow-release Meloxicam (4 mg/kg) was provided during surgery. Animals were provided a minimum of 3 weeks of recovery time before behavioral testing.

### Stimulus Animals

All stimulus animals were gonadectomized and housed in groups of 2–4. At 8–12 weeks of age, stimulus males were castrated and stimulus females were ovariectomized and implanted with a Silastic capsule (1.98 mm id/ 3.17 mm od) containing dissolved 17β-estradiol in sesame oil (50 μg in 0.025 ml) and sealed with Silastic Medical Adhesive Silicone (Dow Corning, Midland, MI, USA). On testing days, stimulus females were injected with progesterone (500 μg in 0.1 ml of corn oil) 2–5 h before testing to induce hormonal estrus (as outlined in Swift-Gallant et al., 2016a). Castrated stimulus males were swabbed on the lower back/near their tail with the urine of sexually experienced male mice (pooled urine of 5–10 male mice; aliquots were frozen at −20°C/thawed on testing days) before each behavioral test. For the olfactory preference test, soiled bedding was collected from sexually experienced males and estrus-induced females 48 h following cage change/induced estrus, and bedding was stored in resealable bags at −20°C. On testing day, bedding reached room temperature before use.

### Behavioral Testing

Mice were between 72 and 90 days of age at the time of testing. Behavioral testing took place between the hours of 12 pm and 4 pm on four separate days with a minimum of 1 day of rest in between tests. The Buried Food test took place on day 1 of testing, Olfactory Preference on day 2, and the Resident-Intruder and the Sexual Behavior test counter balanced between day 3 and day 4 of testing. The terminal odor exposure took place 2–3 days following the final behavior test.

### Buried Food Test

Before testing, mice received a Froot-Loop™ in their cage for three consecutive days to familiarize them with the novel food (following the protocol outlined by Yang and Crawley, 2009). Mice were then food deprived overnight for 18–24 h before testing day. Each animal was first placed in a clean testing cage filled with 3 cm of clean bedding for 5 min of habituation. Mice were then transferred to a holding cage while a Froot-Loop was buried 1 cm under the bedding of the testing cage. The animals were returned to the testing cage and a stopwatch was started. The experimenters stayed in the room (approximately 1 m away) and recorded how long it took the animal to find the Froot-Loop. The time was recorded once the Froot-Loop was retrieved from the bedding and visible to the experimenter. A maximum of 15 min was allotted, however, all animals found the food before time ran out.

### Olfactory Preference

Experimental animals were placed in a clear container (42 cm × 25 cm × 20 cm) with three ramekins containing clean bedding and clean bedding also covered the bottom of the container. Mice were allowed 5 min to habituate before replacing the ramekins with one each of clean, male-soiled, and female-soiled bedding in a quasi-randomized order. The experimenters then left the room and video-recorded a 10-min trial. Videos were later coded by a blind experimenter using Behavioral Observation Research Interactive Software (BORIS) to record the total time spent in each ramekin. Using the testing records, each ramekin was matched with the corresponding bedding type and a female preference score was calculated (time spent in female bedding minus time spent in male bedding).

### Resident-Intruder Paradigm and Sexual Behavior Test

Each animal underwent the resident-intruder test to assess socio-sexual behaviors in response to an intruder male, and a second test to measure sexual behavior in response to an estrus-induced female stimulus mouse, closely following the methodology of Kimchi et al. (2007). Exposure to male or female stimulus mouse was counterbalanced across groups. These tests were conducted in the home cage of the experimental animal with only the bedding remaining along the bottom of the cage (i.e., all enrichment/food was taken out of the cage for testing), and the cage lid was replaced with a clear Plexiglass lid with air holes. Behavior was video-recorded for a 15-min trial while experimenters left the room. Videos were later scored by a blind experimenter using BORIS. Behaviors recorded included anogenital investigation, face/body investigation, self-grooming (genitals), sexual behavior (mounting, thrusting, intromission), and aggressive behavior [attacking (i.e., pounce/lunge towards stimulus animal), tumbling, chasing, boxing, biting]. Note that following olfactory tests, the sample size decreased for resident-intruder paradigm: peripubertal sham *n* = 18; 9 female, peripubertal VNOx *n* = 18; 8 female, adult sham *n* = 20; 9 female and adult VNOx *n* = 19; 9 females.

### Dissections

Ninety-minutes before dissection, mice were exposed to a ramekin filled with opposite-sex soiled bedding for 10 min in their home cage. Mice were weighed then overdosed with Avertin. Mice were perfused with 0.1 M phosphate-buffered saline followed by 4% paraformaldehyde in 0.2 M PBS (PFA). Somatic measures, including the androgen-sensitive tissues Bulbocavernosus/Levator Ani (BC/LA), seminal vesicles, testes, and female uterine horn/ovaries, were weighed. Brains were extracted and post-fixed for 2 h in 4% PFA, then transferred to 20% sucrose. Brains in sucrose were stored at 4°C until sectioning. All olfactory bulbs and brains were coronally sectioned within 2 weeks of dissection on a sliding freezing microtome at 30 μm into four series; tissue was stored in cryoprotectant at −20°C until histological processing. At the time of dissection, the somatic measures were not taken for the first seven animals (one male from peripubertal VNOx, one male from adult sham, one male from adult VNOx, and one female from *each* VNO condition), and thus were excluded from statistical analyses for these measures.

### Immunohistochemistry

One series of four of brain tissue was stained for FOS immunoreactivity (ir), a marker of neural activity. Tissue was washed five times for 5 min in 1× phosphate-buffered saline (PBS), then incubated for 10 min in 0.3% hydrogen peroxide in PBS. Next, with three 5-min washes between each subsequent step, tissue was blocked in 10% normal goat serum (NGS) in PBS-GT (0.3% Gelatin, 0.1% Triton 100 in 1× PBS). Tissue was washed before being incubated in primary (Cell Signaling CAT#2250; 1:10,000) in 1% NGS in PBS-GT for 18–24 h at 4°C. Next, tissue was incubated in 1:500 secondary solution (Goat Anti-Rabbit Biotinylated; Vector Labs, CAT# BA-1000) in PBS-GT for 1-h, then placed in ABC for 1-h (Vector Labs, CAT# PK-6100; two drops of each A and B in 10 ml of PBS). Tissue was then stained in a 3,3′-diaminobenzidine (DAB) nickel reaction for 3–4 min. Brain sections were mounted onto gelatin-subbed slides, dehydrated and coverslipped with Fisher Permount Mounting Medium (Cat# SP-15-500).

Brain regions accessed for FOS-ir included the medial preoptic area (MPOA), bed nucleus of the stria terminalis (BNST), medial amygdala posterior dorsal division (MePD), and ventromedial hypothalamus ventrolateral division (VMH). Given the role of the nucleus accumbens (NAcc) in sexual motivation and the disruption in dopamine in the NAcc among *Trpc2−/−* mice, we also evaluated neural activity (FOS-ir) in this brain region. Bilateral images of two sections for each brain region were taken at 40× magnification; brain regions were identified using the Paxinos and Franklin’s (2019) Atlas, as previously described (Swift-Gallant et al., 2016a).

Images of the FOS-ir stained brain regions were captured using an Olympus bright-field microscope. Bilateral images were taken at the 40× objective of two brain sections for each of the five brain regions for FOS. FOS-ir was measured with the automated cell count function as previously described (Swift-Gallant et al., 2016a).

### Statistical Analyses

Sex and VNO condition effects were assessed for somatic measures, behavioral tests, and FOS-ir, using analysis of variance (ANOVA). Differences in neuroanatomical measures (cell number and size) among VNO conditions were assessed for male subjects only using one-way ANOVAs. Significant omnibus effects were followed with Tukey *post hoc* analyses. Sham-operated animals that underwent the procedure in peripuberty or adulthood did not differ on any measure and thus were collapsed for all statistical tests. Alpha was set at *p* < 0.05.

## Results

### VNO Ablation

VNOx and sham surgeries were confirmed at the time of dissection. All nasal cavities were clear of any debris/blood clots, indicating that all animals recovered from the VNO surgery. All mice from peripubertal or adult VNOx groups were found to have partial or complete VNO ablation at the time of dissection, while those from the sham-operated group had intact VNOs (see Supplementary Figure 1 for a representative image of intact, partial ablation and complete ablation, and Supplementary Materials for statistical analyses with partial VNOx mice excluded).

### Somatic Measures

The expected sex difference was found in body weight, with males weighing more than females, *F*_(1,65)_ = 75.51, *p* < .001, but no differences were found between VNO conditions, *F*_(2,65)_ = 2.37, *p* = .1, nor was there a significant interaction between sex and condition, *F*_(2,65)_ = 1.08, *p* = .35 (Table 1).

**Table 1:** Somatic measures by sex and vomeronasal organ (VNO) condition: Mean weight in grams (SEM).

Sex	VNO condition	Weight	Testes	Seminal vesicles	BC/LA	Uterine horns	Ovaries
Male	Shams	26.3 (0.41)	0.150 (0.004)	0.274 (0.008)	0.102 (0.008)	-	-
	Peripubertal VNOx	26.4 (1.13)	0.162 (0.004)	0.287 (0.02)	0.116 (0.01)	-	-
	Adult VNOx	26.8 (1.01)	0.144 (0.005)	0.309 (0.015)	0.160 (0.074)	-	-
Female	Shams	20.5 (0.55)	-	-	-	0.08 (0.007)	0.0130 (0.002)
	Peripubertal VNOx	20.6 (0.3)	-	-	-	0.078 (0.012)	0.0168 (0.004)
	Adult VNOx	22.9 (0.86)	-	-	-	0.073 (0.011)	0.0111 (0.001)

Overall, the analysis of androgen-sensitive somatic measures suggested that VNO condition did not obviously alter circulating androgens. Males across conditions did not significantly differ in BC/LA, *F*_(2,34)_ = .85, *p* = .44, or seminal vesicle weight, *F*_(2,34)_ = 1.83, *p* = .18. A main effect of condition was trending for testes weight, *F*_(2,34)_ = 3.09, *p* = .058, although *post hoc* tests did not reveal any significant or marginal differences between VNOx groups and sham males (*p*s > .1). In female mice, ovary and uterine horn weight did not significantly differ between VNO conditions, *F*_(2,30)_ = 1.1, *p* = .35 and *F*_(2,30)_ = .16, *p* = .85, respectively.

### Buried Food Test

**Figure 2:**
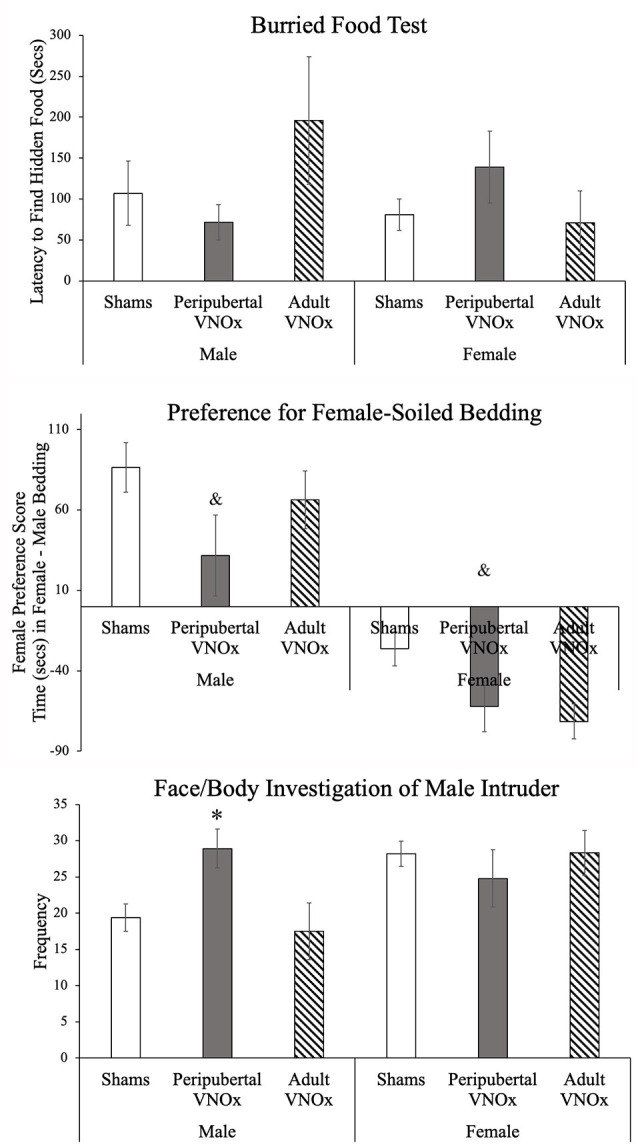
Olfactory tests, Mean ± SEM. Groups did not differ on the Buried Food test, and all animals found the hidden food in the allotted time (top panel). On the Olfactory Preference test, both peripubertal and adult VNO ablation decreased preference for female soiled-bedding (middle panel). In the Resident Intruder paradigm, males with peripubertal, but not adult, VNO ablation showed an increase in the investigation of the face/body of the male intruders; no differences were found between female VNO condition on this measure. ^&^Indicates a main effect of VNO condition and significant *post hoc* test when compared to sham animals, *indicates a significant difference from other same-sex groups, *p* < .05.

All experimental mice were successful in uncovering the hidden food in the allotted time (Figure 2). We found no differences between groups in latency to find the hidden food: main effect of sex: *F*_(1,77)_ = .66, *p* = .42, main effect of VNO condition: *F*_(2,77)_ = .46, *p* = .62, and interaction sex by condition: *F*_(2,77)_ = 2.13, *p* = .13.

### Olfactory Preference

We found the expected sex difference on the olfactory preference test, such that male mice displayed a greater female preference score compared to female mice, *F*_(1,77)_ = 58.71, *p* < .001. While the sex by VNO condition interaction was not significant, *F*_(2,77)_ = .16, *p* = .85, a main effect of VNO condition was found, *F*_(2,77)_ = 4.92, *p* = .01, such that mice with peripubertal VNOx showed a decreased female preference score compared to sham-operated animals, *p* = .009. Mice with adult VNOx did not differ from peripubertal VNOx or sham mice (all *p*’s > .1). These results suggest that peripubertal VNOx altered olfactory preference in mice, whereas adult VNOx led to an intermediate phenotype, such that they did not differ from sham-controls or peripubertal VNOx mice (Figure 2).

### Anogenital and Face/Body Investigation of Estrus Female Intruders and Male Intruders

Anogenital investigation of a female intruder (frequency and duration) did not differ by sex, VNO condition, or sex by VNO condition (all *p*s > .1). A main effect of sex was found for number of face/body investigations of a female intruder, such that female experimental mice did this behavior more than male mice, *F*_(1,69)_ = 4.01, *p* = .049; no VNO condition or condition by sex effects were found for this measure, *F*_(2,69)_ = .57, *p* = .57 and *F*_(2,69)_ = 1.08, *p* = .35, respectively. No significant sex, VNO condition, or sex by condition effects were found for the duration of face/body investigation of a female intruder (Table 2).

**Table 2:** Sexual behaviors in response to an estrus female: mean (SEM).

		Face/body investigation frequency	Anogenital investigation frequency	Latency to mount (secs)	Mount frequency	Thrust frequency	Intromission frequency	Self-groom frequency	Self-groom duration
Male	Shams	22.6 (1.81)	12.0 (1.51)	545 (74.6)	12.1 (2.02)	26.4 (6.38)	38.4 (14.1)	5.15 (1.16)	40.5 (12.3)
	Peripubertal VNOx	26.4 (3.77)	13.1 (2.01)	530 (95.8)	9.40 (3.52)	27.0 (10.1)	34.1 (12.5)	6.20 (2.35)	63.4 (23.4)
	Adult VNOx	22.3 (4.16)	10.7 (1.55)	373 (89.7)	18.4 (4.74)	37.3 (12)	64.3 (32.5)	8.10 (4.28)	47.1 (23)
Female	Shams	26.7 (2.78)	11.9 (1.28)	651 (74)	3.39 (1.35)	7.11 (3.28)	11.4 (6.57)	0.333 (0.23)	1.62 (1.21)
	Peripubertal VNOx	27.1 (2.67)	13.0 (1.84)	646 (125)	2.00 (1.05)	3.00 (2.18)	1.75 (1.37)	0.00	0.00
	Adult VNOx	32.7 (2.39)	10.3 (1.05)	615 (91.6)	2.56 (1.23)	1.56 (0.97)	0.222 (0.22)	0.00	0.00

Conversely, in response to a male intruder, we found a significant sex by condition effect for number and duration of face/body investigation of a male intruder, *F*_(2,69)_ = 3.8, *p* = .027 and *F*_(1,69)_ = 3.3, *p* = .043, respectively. *Post hoc* tests indicated that males with peripubertal VNOx did not differ from females (sham or ablation groups) in number or duration of face/body investigation of a male intruder (*p*s > .1), whereas sham and adult VNO ablation males performed this behavior *less* than sham females (*p*s < .05). Female groups did not significantly differ from each other (*p*s > .1). Together, these results suggest that peripubertal VNO ablation in males increases the face/body investigation of male intruders.

An effect of sex was found for the number of face/body investigation of a male intruder, *F*_(1,69)_ = 5.25, *p* = .025, indicating that overall females showed an increase in the investigation of male intruders than male experimental mice; the main effect of sex was not significant for the duration of face/body investigation of the male intruder, *F*_(1,69)_ = .08, *p* = .79.

No sex, condition, or sex by condition effects was found for the number or duration of anogenital investigations of a male intruder (*p* > .1).

### Sexual Responses to Estrus Female Intruder and Male Intruder

VNO condition did not affect sexual behaviors in response to a male or female intruder, though we found the expected effect of sex on most measures. Specifically, male mice showed a lower latency to mount, and they mounted, thrusted, intromitted, and self-groomed more than female mice in response to a female stimulus mouse (main effects of sex, *p*s < .05; Table 2).

### Aggressive Responses to Male Intruder

**Figure 3:**
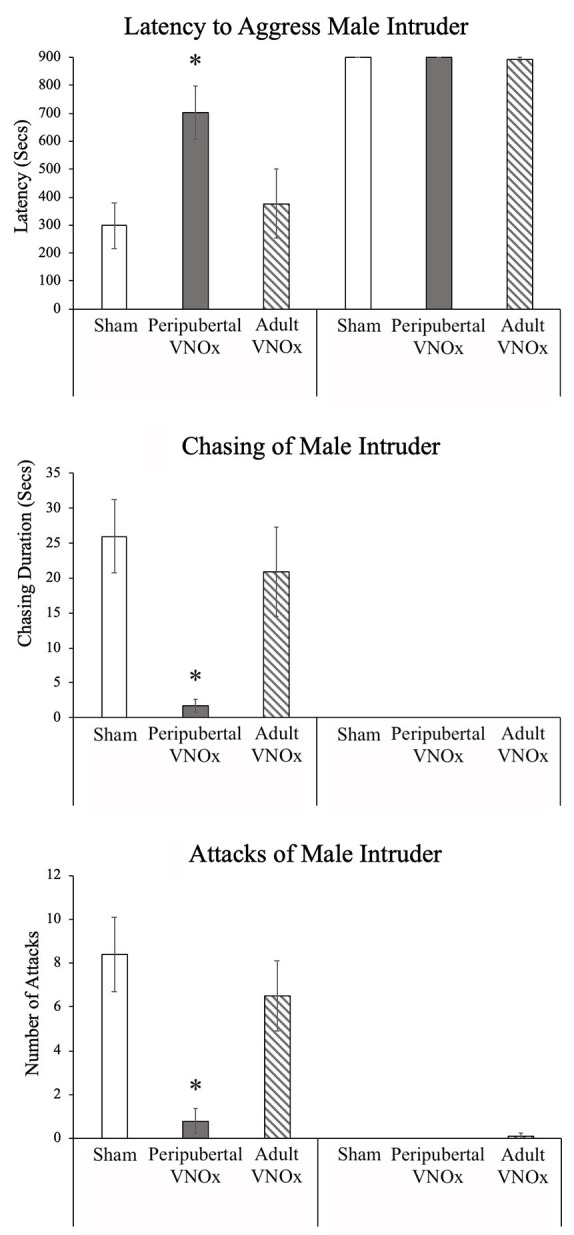
Aggressive behavior in response to a male intruder, Mean ± SEM. Peripubertal VNOx resulted in a reduction in inter-male aggressive behaviors among male mice. Latency to aggress is longer, and chasing and attacking (i.e., lunge/pounce) towards a male intruder is significantly reduced in males with peripubertal VNO ablation, whereas adult VNOx males do not show any impediments in territorial aggression when compared with sham-operated control males. *Indicates a significant difference from other same-sex groups, *p* < .05.

We found the expected sex difference in aggressive behavior, such that males displayed more chasing, attacks, biting, and tumbling compared to females in response to a male intruder (*p*s < .05). We also found that peripubertal, but not adult, VNOx led to a decrease in aggressive behaviors. Specifically, significant interactions of sex and VNO condition were found for latency to aggress (i.e., latency to show any aggressive behavior), *F*_(1,69)_ = 3.62, *p* = .032, number and duration of chasing of male intruders, *F*_(2,69)_ = 3.51, *p* = .035 and *F*_(2,69)_ = 4.36, *p* = .016, as well as for number of attacks (i.e., lunge/pounce) towards a male intruder, *F*_(1,69)_ = 4.5, *p* = .015. *Post hoc* tests indicated that peripubertal VNOx males displayed a decrease in all of these aggressive measures compared to both sham and adult VNOx males (*p*s < .05), whereas female VNO conditions did not differ from one another (*p*s > .1; Figure 3). No effects of VNO condition were found for tumbling, biting, or boxing, although it should be noted that these behaviors were rarely observed in all groups (i.e., only six animals showed any biting or boxing).

### Neural Activity in Response to an Opposite-Sex Odor

**Figure 4:**
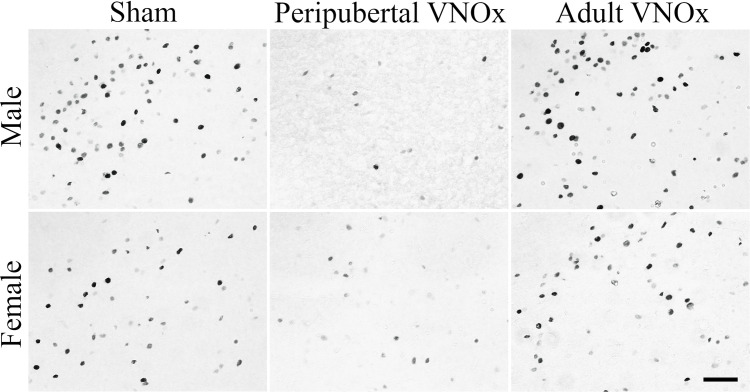
Representative images of FOS-ir in the medial amygdala posterior-dorsal (MePD) division. Overall, mice with Peripubertal VNOx showed a reduction in FOS-ir in the NAcc, VMH, and MePD in response to an opposite-sex odor when compared with Sham and Adult VNOx mice. Scale bar = 50 μm.

**Figure 5:**
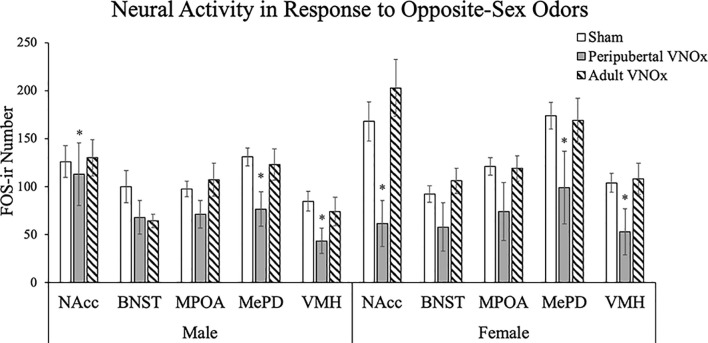
Mean ± SEM FOS immunoreactive (FOS-ir) cell counts in response to an opposite-sex odor. Mice with peripubertal, but not adult, VNOx showed a reduction in neural activity in response to an opposite-sex odor in the NAcc, VMH, and MePD. *Indicates a significant main effect of VNO condition, *p* < .05.

To account for multiple comparisons in the following analyses (i.e., five ANOVAs, one for each brain region), a Bonferroni correction was applied (i.e., alpha was set at *p* < .01 for the following comparisons). FOS-ir analyses suggest that peripubertal, but not adult, VNOx alters the neural response to opposite-sex odors (Figures 4, 5). Specifically, a main effect of VNO condition was found for the NAcc, *F*_(2,59)_ = 4.92, *p* = .01, VMH, *F*_(2,59)_ = 5.64 *p* = .006, and MePD, *F*_(2,59)_ = 6.29, *p* = .003. The main effect of VNO condition did not reach significance in the MPOA, *F*_(2,59)_ = 3.87, *p* = .027 and BNST, *F*_(2,59)_ = 2.16, *p* = .12. Tukey *post hoc* tests indicated that peripubertal VNOx mice displayed reduced FOS-ir in the NAcc, VMH, and MePD compared to both shams and adult VNOx mice (*p* < .05).

The main effect of sex did not reach significance in the NAcc, *F*_(1,59)_ = .73, *p* = .398, MPOA *F*_(1,59)_ = 1.06, *p* = .31, BNST, *F*_(1,59)_ = 0.34, *p* = .57, VMH, *F*_(1,59)_ = 3.00, *p* = .088 and MePD, *F*_(1,59)_ = 5.59, *p* = .021. The interaction of sex and VNO condition was not significant for any brain region.

## Discussion

The present study evaluated the effects of surgical ablation of the VNO during the peripubertal period vs. adulthood on socio-sexual behavior in mice. Our results suggest that puberty is a critical window for VNO mediation of socio-sexual behavior. Peripubertal VNO ablation decreased both preferences for female bedding and neural activity along the vomeronasal-accessory olfactory neural pathway in response to opposite-sex odors. In males, peripubertal VNO ablation increased pro-social behaviors towards a male intruder, such as face and body investigation, while decreasing inter-male territorial aggression. Conversely, adult VNO ablation only had subtle effects on sexual odor preferences in male mice, while socio-sexual behaviors in response to male or female live stimulus mice remained unaltered. These results indicate that the VNO is not merely required for the identification of and/or attraction to conspecifics during an encounter, but instead, a functional VNO in the pubertal critical period has long-lasting effects on the display of sex-typical behavior in male mice.

The finding that peripubertal VNO ablation results in decreased inter-male territorial aggression are consistent with literature identifying puberty as a critical organizational period for male aggression (Romeo, 2003; Schulz and Sisk, 2016). For example, aggressive behavior in male Syrian hamsters, gerbils, and mice is dependent upon androgens during puberty (Schulz and Sisk, 2016). Our findings are also consistent with research demonstrating that disabling the VNO *via* genetic approaches (TRPC-KO) reduces aggressive behavior in male mice (Stowers et al., 2002). The present study sits at the intersection of these two lines of research, demonstrating that the organization of aggressive behavior during the pubertal period is VNO-dependent. Future work may consider *how* the removal of the VNO in the early peripubertal period alters adult behavior. For example, it may be that the pubertal androgen surge can act in part *via* the VNO to organize the brain to promote adult inter-male aggressive behavior. Neonatal work suggests that the neonatal VNO is sensitive to androgens (i.e., sexual differentiation of the VNO is androgen-dependent; Segovia and Guillamón, 1993; reviewed in Monks and Swift-Gallant, 2018), thus puberty may be a second critical period upon which androgens act on the VNO, which may affect the differentiation of the brain and behavior. It is also possible that a non-functional VNO may alter the hypothalamic-pituitary-gonadal axis, leading to alterations in circulating androgens. We did not find any differences between VNO conditions in somatic measures that are androgen-sensitive, however, it remains a possibility that VNO ablation in the peripubertal period could affect the pubertal surge in androgens. Alternatively, it could be the lack of pheromone processing by the VNO that alters the organization of the brain and behavior, permanently altering aggressive behavior in peripubertal VNOx males. However, since all experimental animals in the current study were singly housed in the early peripubertal period (28–36 days of age), these mice were largely only exposed to their own pheromones during the pubertal period. Although it is also possible that they were exposed to opposite-sex and same-sex volatile odors during weekly cage change. Future work may consider whether androgen action *via* the VNO and/or pheromonal input during puberty promotes later inter-male aggression.

We also considered how ablating the VNO during peripuberty is permanently altering behavior at the cellular and circuit levels. Since adult VNOx alone does *not* alter aggression, it appears that it is not merely the incapacity to process pheromones at that time of the interaction with conspecifics that mediate socio-sexual behavior, but rather the lack of a VNO during puberty permanently alters the behavioral response. One possibility is that the VNO disruption at puberty is changing the structure or function of the connected neural circuit. Our results support this in part as we observed changes in FOS-ir in the accessory olfactory pathway in peripubertal VNOx, but not adult VNOx, mice, although we did not observe structural differences (cell size or number) between groups (see Supplementary Materials). These results suggest that the lack of a VNO in puberty alters the responsiveness of the NAcc, VMHvl, and MePD to opposite-sex odors.

FOS immunoreactivity along the accessory olfactory pathway in response to opposite-sex odors often coincides with a preference for opposite-sex odors (e.g., Bodo and Rissman, 2008; Swift-Gallant et al., 2016a), including in the present study, such that peripubertal VNOx mice show both a reduction in female preference score on the olfactory preference test and a decrease in FOS-ir in the NAcc, VMHvl, and MePD. In contrast, adult VNOx males displayed subtle differences on the olfactory preference test, such that they did not differ from sham or peripubertal VNOx mice, although they did not show any differences in FOS-ir compared to sham mice. However, prior work has indicated a decrease in both sexual odor preferences and FOS in these brain regions in adult VNOx males. For example, Samuelsen and Meredith (2009) reported a decrease in FOS-ir in the MePD of adult VNOx males in response to female odors as well as in response to hamster vaginal fluid and worn cat collar. Pankevich et al. (2004, 2006) also reported both a decrease in preference for female odors and a decrease in a marker of neural activity along the accessory olfactory pathway in adult VNOx males in response to female estrous urine. Keller et al. (2006) tested adult VNOx investigation of various volatile and non-volatile odors and found that overall, adult VNOx mice maintained an olfactory preference for volatile (i.e., urinary odors) but did not display a preference for non-volatile odors (soiled-bedding and non-volatile urinary odor). One possibility for the differences between prior work and the current study is the type of odor stimulus used. In the present study, mice had access to soiled bedding in both the olfactory preference test and terminal odor exposure, which likely contains various pheromonal cues of volatile and non-volatile nature, including salivary, urinary, and fecal. Access to a wide spectrum of odors could have masked the deficits in adult VNOx mice in our paradigm.

Alternatively, sexual experience may have masked deficits to some degree in adult VNOx mice. In the present study, mice had a sexual experience before the terminal odor exposure, whereas mice were sexually naïve in the prior studies aforementioned. Sexual experience with a female may have been rewarding which may have resulted in an association between female odors processed by the MOE/MOB and sexual reward (i.e., see Kang et al., 2006 for work showing that odor stimuli processed by MOB are sent to the MePD); however, this was not the case in peripubertal VNOx males, suggesting that sexual experience was not sufficient to make this association/overcome the deficits in neural response to female odors. On the other hand, adult VNOx males in the present study did show an intermediate phenotype on the olfactory preference test, suggesting subtle deficits, and thus, another possibility for the contrasting results is that the FOS-ir analyses were not sensitive enough in the present study to discern subtle effects of adult VNOx. Thus, while adult VNOx mice may have subtle differences in odor preferences and FOS-ir in response to sexual odors, the results of the present study support the hypothesis that the pubertal VNO plays a critical for the sexual differentiation of the brain and behavior.

Previous research has indicated that both surgical and genetic disruption of the VNO alters the nucleus accumbens core, part of the mesolimbic dopamine pathway (Pankevich et al., 2006; Beny-Shefer et al., 2017), and the NAcc was one brain region that showed decreases in FOS-ir in peripubertal VNOx mice. Thus, future work may also consider whether the pubertal VNO contributes to the differentiation of the dopaminergic pathway or receptors in the accessory olfactory pathway/NAcc and whether such differences in the NAcc contributes to the deficits in male territorial aggression and odor preferences observed in the current study.

Adult or peripubertal VNO ablation did not alter the display of socio-sexual behavior of females in response to live stimulus animals. This is contradictory to the drastic reversal in sexual behaviors reported in *Trpc2*^−/−^ females or adult surgically ablated females, which was reported by Kimchi et al. (2007) to indicate the existence of a functional neural-circuit that supports male-typical behavior in females that is normally suppressed by the VNO. However, Martel and Baum (2009) report that adult VNO ablation is insufficient to produce male-typical sexual behavior in female mice, which is also reflected in our results. Instead, our results indicate that the pubertal period is a critical window upon which a functional VNO is required for male-typical aggression in adulthood. The earlier neonatal period may also be a critical period for VNO mediation of sexual behavior—this would in part account for the discrepancies between the genetic knockout (*Trpc2*^−/−^; Kimchi et al., 2007) and surgical ablation studies (i.e., the current study and Martel and Baum, 2009). Alternatively, discrepancies between the current study/adult VNO ablation studies and the *Trpc2*^−/−^ experiments may be due to the non-specificity of the genetic ablation model—for instance, *Trpc2* expression has been reported in the MOE (Omura and Mombaerts, 2014; reviewed in Yu, 2015). Nevertheless, our study together with Baum’s work contradicts the proposal of Kimchi et al. (2007)—the adult VNO does not seem to be suppressing male-typical behavior, but rather the present study indicates that the pubertal VNO can have long-lasting effects on the sexual differentiation of behavior.

In contrast to the current study, Clancy et al. (1984) reported that adult surgical VNOx decreases male sexual behavior and territorial aggression. However, the behavioral effects observed in Clancy et al. (1984) were subtle, and there are notable methodological differences to consider. First, Clancy et al. (1984) reported that in response to a receptive female, adult VNOx males did not differ from sham males in latency, though they did show a reduction in the frequency of mounts and intromissions. Notably, their test length was 60 min whereas, in the present study, we only evaluated a 15-min interaction with a receptive female. Thus, it is possible that with an increase in test length, subtle differences in male sexual behaviors may have emerged in the adult VNOx males in the present study.

Clancy et al. (1984) also reported that adult VNOx impaired male territorial aggression. However, the effects of VNOx on aggression were also subtle. Adult VNOx males in their study showed an equivalent frequency of aggressive behaviors to sham males, and adult VNOx males also did not differ from sham males in latency to aggress; the difference emerged in their analysis of the number of animals that showed aggression. With such analyses, one could argue that a mild effect of adult VNOx could be found in the present study—three out of 17 sham males, three out of 10 adult VNOx males, and eight out of 10 peripubertal VNOx males did *not* show any aggression in response to stimulus males. A Kruskal Wallis on this data, followed with DSCF pairwise comparisons, indicates that the number of males showing aggression is significantly decreased in peripubertal VNOx males than sham males (*W* = −4.84, *p* = 0.002), while adult VNOx males are intermediate, such that they do not significantly differ from either group (*W* = −1.35, *p* = 0.61 and *W* = 3.1, *p* = 0.073, respectively). Thus, adult VNOx may have subtle effects on territorial aggression, however, the effects of peripubertal VNOx are greater and more consistent across animals, suggesting a critical role of the pubertal VNO for male territorial aggression.

Similar to the present study, Clancy et al. (1984) did *not* show an increase in mounting in response to a stimulus male, which is in contrast to genetic ablation studies, where Trpc2-KO male mice displayed high levels of sexual behavior towards same-sex conspecifics (Leypold et al., 2002; Stowers et al., 2002). Altogether, the present study in conjunction with prior work, suggests that adult VNOx may have a subtle effect on sexual behavior, but the early neonatal VNO may have an effect on same-sex directed sexual behavior in mice.

### Limitations

While we closely followed the methodology of Kimchi et al. (2007) to evaluate the effects of VNOx on male sexual behaviors in response to both an estrus female and castrated male swabbed with the urine of sexually experienced males, we did not evaluate female receptivity in experimental animals. Namely, we did not assess the estrus cycle of experimental female mice and we did not present mice with a sexually experienced male; thus, the present study cannot address whether peripubertal or adult VNOx affects female sexual behavior.

Other limitations of the current study include the time of behavior testing—due to facility limitations, mice were tested during the light cycle. While mice are more likely to display behaviors in the dark cycle when they are most active, we did see comparable levels of sexual behavior to studies conducted in the dark cycle (e.g., Swift-Gallant et al., 2016b reported a mean of 7.3 mounts and 10 thrusts by unmanipulated wildtype male in response to stimulus females, while in the current study sham males displayed a mean of 12.1 mounts and 26.4 thrusts; Table 2), and prior work on VNOx, such as Clancy et al. (1984), also measured sexual and aggressive behaviors in the light cycle.

While the goal of the present study was to ablate the VNO in peripuberty before the major endocrine events, the onset of puberty can vary between individual animals, and thus it is possible that some mice were further along in their development than others. Future work may consider using a marker, such as verification of vaginal opening or balano-preputial separation in each animal, to manipulate the VNO at the same developmental period across experimental mice.

Lastly, we included mice with partial ablation of the VNO in the principal analyses; we did so, because in all cases the posterior portion of the VNO, which connects the organ to the olfactory bulbs, was destroyed, thus it was likely rendered non-functional. However, to rule out the possibility that this impacted the results, we provide the statistics comparing groups with partial VNOx mice excluded from the analyses in the Supplementary Materials—by in large, the findings are similar, suggesting that partially ablated VNOx resulted in a non-functional VNO.

Altogether, the present study further elucidates the role of the peripubertal VNO and adult VNO for socio-sexual behavior in mice, however, future work may consider these methodological limitations in their experimental design.

### Conclusions

The present study suggests that the sex-typical development of socio-sexual behavior in mice is VNO-dependent. Peripubertal ablation of the mouse VNO decreased both preferences for female odor and neural activity along the vomeronasal-accessory olfactory pathway in response to opposite-sex odors in both males and females. In males, peripubertal VNO ablation also decreased territorial aggression in response to a male intruder and increased pro-social behaviors, such as face/body olfactory investigation. Conversely, adult VNO ablation largely did not impact behavior or brain activity in response to opposite-sex odors, though had a subtle effect on sexual odor preference. Together, these findings suggest that puberty is a critical period for the VNO mediation of socio-sexual behavior, while in adulthood the VNO plays a more subtle role in odor preferences. Future work may consider whether the VNO in early neonatal periods of development plays a role in the development of socio-sexual behaviors, especially sexual behavior, given that alterations in copulatory behavior have been reported in lifelong genetic VNO ablation models.

## Data Availability Statement

The raw data supporting the conclusions of this article will be made available by the authors, without undue reservation.

## Ethics Statement

The animal study was reviewed and approved by Memorial University of Newfoundland Animal Care Committee.

## Author Contributions

SC and AS-G were responsible for the experimental design and writing of the first draft of the manuscript. All authors contributed to the editing of the manuscript. SC, YM, SS, IG, CM, SH and KP contributed to the data collection and data analysis. AS-G acquired funding to support the collection of data and student stipends. All authors contributed to the article and approved the submitted version.

## Conflict of Interest

The authors declare that the research was conducted in the absence of any commercial or financial relationships that could be construed as a potential conflict of interest.
