# Supplementary material for: Puberty is a Critical Period for Vomeronasal Organ Mediation of Socio-sexual Behavior in Mice
Source: Front Behav Neurosci. 2021 Jan 22;14:606788. doi: 10.3389/fnbeh.2020.606788 (PMC7862124; doi:10.3389/fnbeh.2020.606788)
Supplement: Supplementary file 1 [file Data_Sheet_1.doc]

Supplementary Materials

*Peripubertal vs Adult Sham Mice*

We included sham-operated controls at both time points, peripuberty and adulthood. However, because these two groups did not differ on any measure, we collapsed these groups for the analyses presented in the main manuscript. In this section, we present the statistics comparing peripubertal and adult sham mice on focal measures.

Peripubertal and adult sham males did not differ in testes, seminal vesicle or BC/LA weight (all *p* < .1). Body weight also did not differ by surgery timing (peripubertal vs adult sham operation), *F*(1, 32) = 1.56, *p* = .22, nor was there an interaction of sex and surgery timing on body weight, *F*(1, 32) = 0.09, *p* = .76.

On the buried food test and female preference score, the effect of surgery timing and interaction of sex and surgery timing were non-significant, *F*(1, 38) = 0.02, *p* = .90, *F*(1, 38) = 1.74, *p* = .19, *F*(1, 38) = 2.32, *p* = .17 and *F*(1, 38) = 0.15, *p* = .70, respectively. Similarly, in response to a male intruder, no main effects of surgery timing or interaction between surgery timing and sex, were found on frequency face/body investigations (*F*(1, 34) = 1.96, *p* = .17 and *F*(1, 34) = 0.29, *p* = .59), chasing duration (*F*(1, 34) = 0.04, *p* = .84 and *F*(1, 34) = 0.04, *p* = .84) and attacks (*F*(1, 34) = 0.06, *p* = .81 and *F*(1, 34) = 0.06, *p* = .81).

*Partial vs Complete VNO Ablation*

Mice with partial ablation of the VNO were included in the present study, as in all cases, the most posterior portion, which connects the organ to the olfactory bulbs, was destroyed (see Fig S1, Panel B), and excluding these mice greatly reduces the sample sizes (peripubertal VNOx mice n = 13; 6 males, adult VNOx mice n = 15; 8 males). Nevertheless, we conducted the following analyses excluding mice with partial ablation to assess whether the overall pattern of results would remain similar when comparing mice with complete VNOx and sham-operated mice.

On the buried food test, no significant main effects or interactions were found (*ps* > .1). On the olfactory preference test, the main effects of sex and VNO condition were significant, *F*(1, 70) = 56.7, *p* < .001, and *F*(2, 70) = 54.04, *p* = .02, respectively. Tukey post hoc comparisons indicated that peripubertal VNOx mice had a reduced female preference score compared to sham-operated controls (*p* = .02), but adult VNOx mice did not differ from peripubertal VNOx (*p* = .54) or sham controls (*p* = .33).

In response to a male intruder, the sex by VNO condition interaction on frequency of face/body investigations remained significant when excluding mice with partial VNOx, *F*(2, 59) = 3.34, *p* = .042, as well as the main effect of sex, *F*(1, 59) = 9.04, *p* = .004. Sex by VNO condition interactions were found for duration of chasing, *F*(2, 59) = 3.22, *p* = .047, and was trending for frequency of attacks, *F*(2, 59) = 2.88, *p* = .064. Post hoc analyses on these measures of aggression indicated that peripubertal VNOx male mice showed a reduction in aggression towards a male intruder compared to both sham males and adult VNOx males (*p* = .023-.06); peripubertal VNOx males did not differ from female groups (*p* = .98-1), whereas sham males and adult VNOx males differed from all female groups (*p* = .001-.01).

*Neuroanatomy of the LS, MePD, VMHvl and AOB in Peripubertal and Adult VNOx Mice*

Pilot analyses were conducted to assess whether peripubertal and/or adult VNO ablation resulted in gross neuroanatomical differences (Cresyl Violet staining) in brain regions associated with aggression, including the lateral septum (LS), medial amygdala posterior dorsal division (MePD) and ventromedial hypothalamus ventrolateral division (VMHvl) in experimental male mice. Given the primary behavioral differences observed among VNO conditions was in aggressive behavior in male mice (see “Results”), we were interested in evaluating whether cell number or cell size was altered in LS and VMH (i.e., these connected brain regions have been directly related to the display of aggressive behaviors without affecting sexual behavior: e.g., Wong, Wang, D’amour et al., 2016). Furthermore, the MePD receives information directly from the vomeronasal-accessory olfactory bulb and is connected with the VMH. Thus, we hypothesized that if we were to see structural differences in the brains of these mice, it would be within these three brain regions. Note that tissue damage resulted in one less animal (sham male) included in the MePD neuroanatomical analyses. We also assessed the area of the accessory olfactory bulbs (AOB) in a subset of experimental male mice. These pilot analyses focused on male subjects as the greatest behavioral differences were found among males.

Cresyl Violet staining was used to assess neuroanatomical structure among male VNO conditions. One series from four of brain tissue was mounted in 1X PBS on to gel-subbed slides. All slides were stained using a 0.5% Cresyl Violet Nissl stain. They were then dehydrated and coverslipped with Permount. The protocol consisted of ten dips into deionized water, 10 minutes in 0.5% Crestyl Violet, 10 seconds in 70% ethanol with acetic acid (250 mL EtOH and 3 drops of acetic acid), 1 minute in 70% ethanol, 2x2 minutes in 100% ethanol, and 2x2 minutes in Xylene. All cells within the images were manually counted, and 10 neurons (cells with a single nucleolus and tear-drop shape) closest to the center focus of each image were outlined and the area was measured with the Analyze🡪 Measure function in ImageJ. Males from the three VNO condition groups (sham, peripubertal ablation and adult ablation) were compared on the average number and size of cells for each brain region.

We did not find any significant differences between shams, peripubertal and adult VNOx males in the cell number or cell size in the LS, MePD and VMH (all *ps > .1*; Figure S2; Table S1 and S2).

We also conducted a pilot on a subset of animals to access whether peripubertal and/or adult VNOx resulted in gross alterations to the accessory olfactory bulbs (AOB). In the AOB, we measured the area of AOB from 3 sections per animal (n = 6 males/group), as well as the area of the three layers of the AOB (granule, mitral and glomerular; Figure S3), using ImageJ. However, we did not find any evidence of degeneration in any of the three layers of the AOB (Table S3).


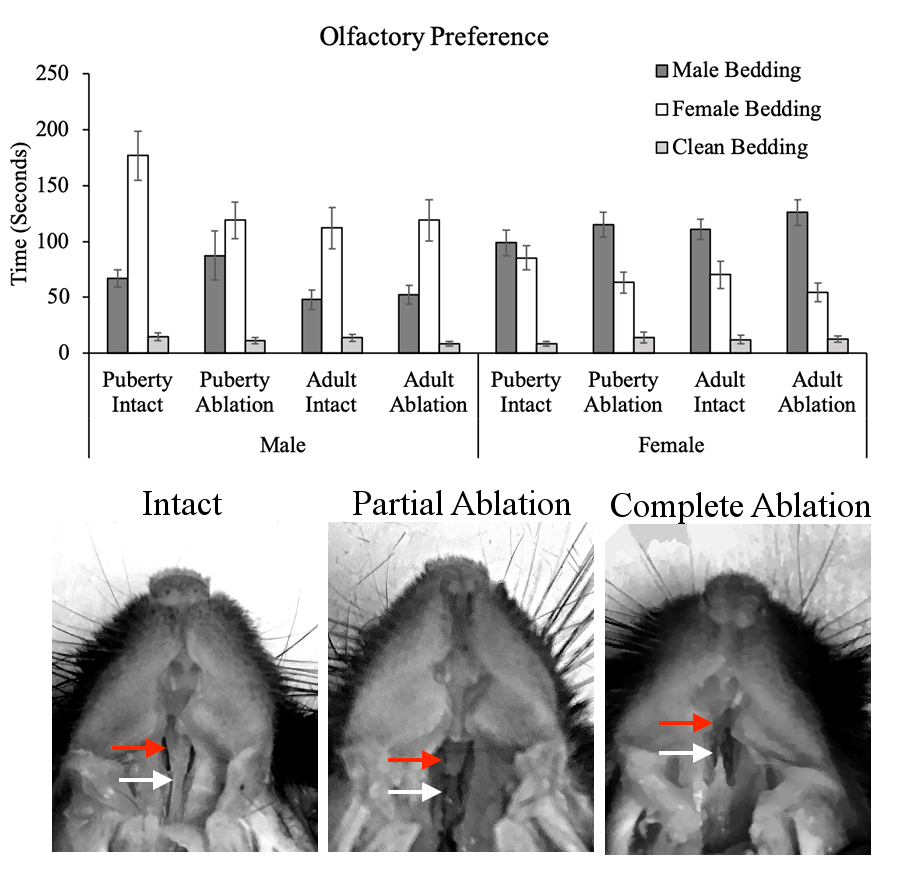


A

B

B

Figure S1. Panel A shows raw data for the olfactory preference test. Note that all animals, regardless of VNO condition, show a preference for soiled-bedding over clean bedding. Panel B shows examples of an intact VNO, partially ablated/disconnected VNO and complete VNO ablation. Adult and pubertal ablation groups did not differ in number of complete ablations and partial ablations, *X^2^* = 0.12, *p* = .73.

*
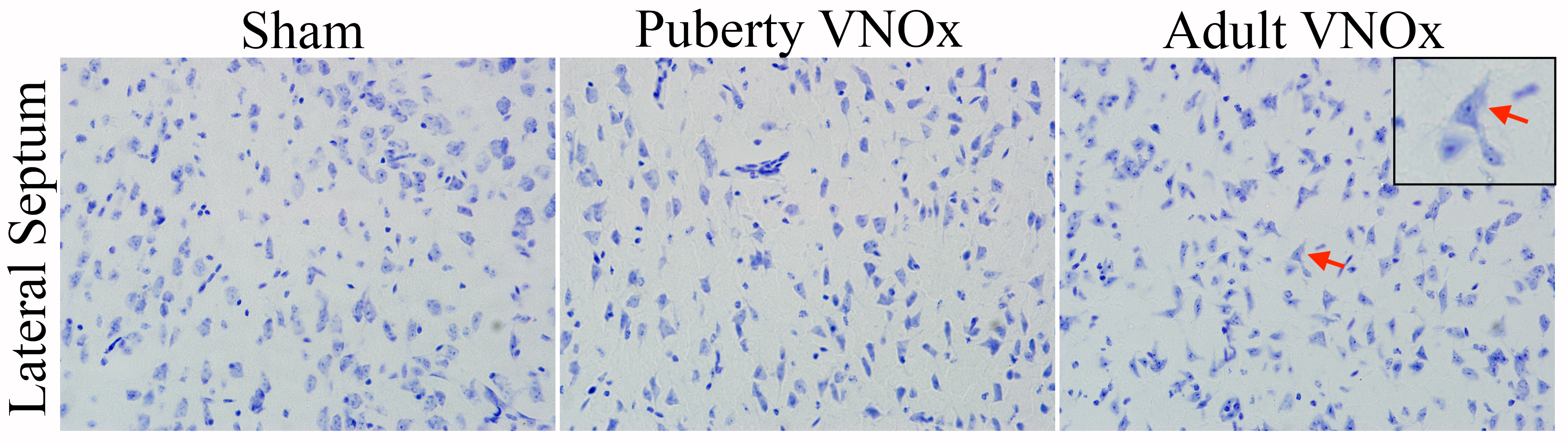
*

Figure S2. *Nissl staining in the Lateral in Peripubertal VNOx, Adult VNOx and Sham males.* Cell number and cell size measurements in the Lateral Septum (LS), Ventromedial Hypothalamus (VMH) and Medial Amygdala Posterior Dorsal division (MePD) were compared across male VNO conditions. Bilateral images of two brain sections per brain region were captured at 40x magnification and ImageJ was used to count all cells within view, and measure the area of ten cells identified as neurons (cells with a single nucleolus; red arrow points to an example), closest to the central point of focus of each image. Results indicate that peripubertal or adult VNO ablation did not alter cell number or size in the LS, VMH or MePD, when compared with sham controls.


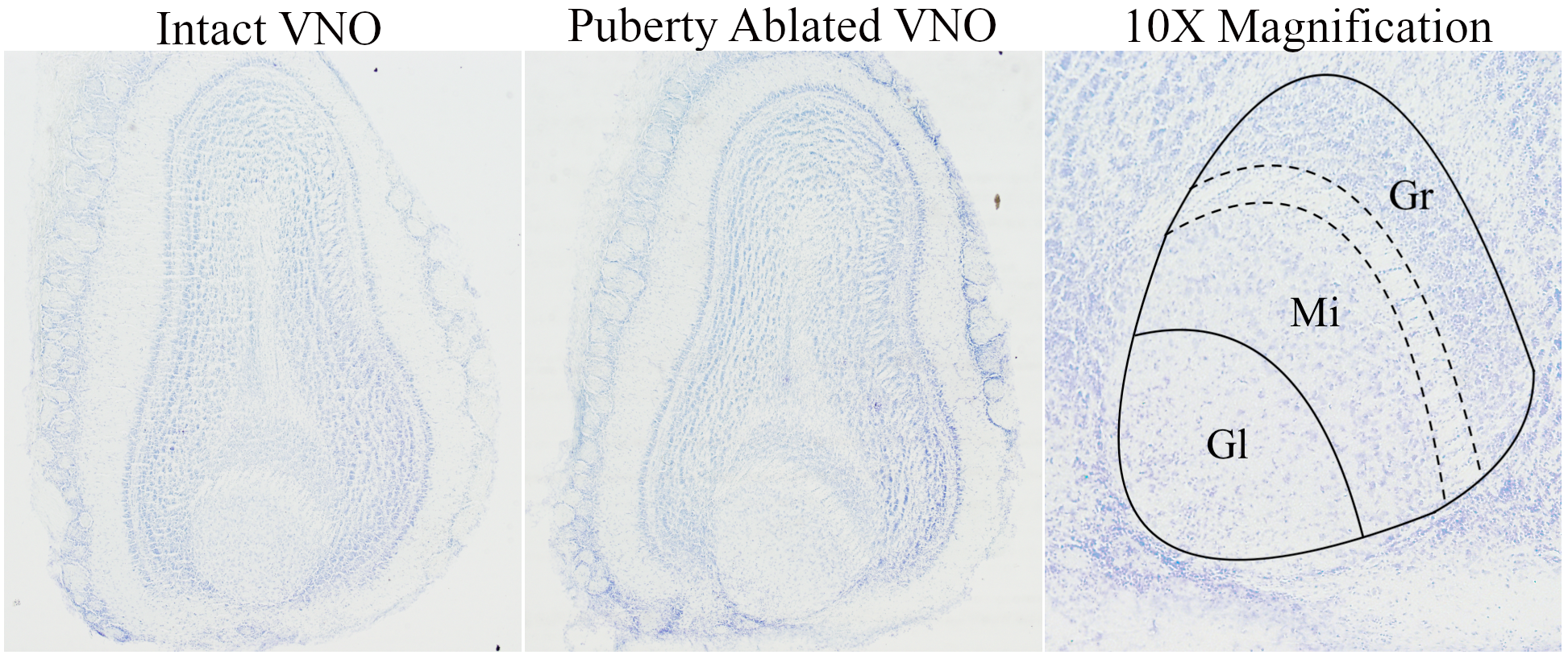


Figure S3. Accessory Olfactory Bulbs in Intact/Sham and Peripubertal VNO Ablated Male Mice. We measured the total area as well as the area of the 3 layers of the accessory olfactory bulb (granule [Gr], mitral [Mi] and glomerular [Gl]) from Nissl stained sections; however, we did not find any evidence of degeneration or a reduction in size of the accessory olfactory bulb in mice with peripubertal or adult VNO ablation compared to sham control mice. *Left and middle panel = 2X magnification, right panel = 10X magnification.*

Table S1. Brain Nissl Analyses: Cell Size in Pixels, Mean (SEM)

| Treatment | LS | MePD | VMHvl |
| --- | --- | --- | --- |
| Sham (n = 15)  Peripubertal VNOx (n = 10)  Adult VNOx (n = 8) | 1236 (49.1)  1174 (57.5)  1159 (74.5) | 1293 (57.2)  1296 (50.5)  1213 (87.3) | 1400 (74.9)  1311 (56.1)  1221 (78.9) |

Table S2. Brain Nissl Analyses: Cell Number, Mean (SEM)

| Treatment | LS | MePD | VMHvl |
| --- | --- | --- | --- |
| Sham (n = 15)  Peripubertal VNOx (n = 10)  Adult VNOx (n = 8) | 398 (27.8)  424 (21.3)  460 (44.3) | 522 (45.1)  505 (16.7)  637 (143) | 519 (47.5)  523 (28.3)  670 (20.3) |

Table S3. Nissl Analysis of Accessory Olfactory Bulbs: Area in Pixels, Mean (SEM)

| Treatment | Granule | Mitral | Glomerular | Total Area |
| --- | --- | --- | --- | --- |
| Sham (n = 6) | 2006 (262) | 587 (31.3) | 1114 (30.3) | 3848 (268) |
| Peripubertal VNOx (n = 6) | 2039 (266) | 513 (43.8) | 1069 (63.7) | 3829 (342) |
| Adult VNOx (n= 6) | 2160 (170) | 531 (46.4) | 1145 (63.3) | 4066 (241) |
